# Effect of Tiotropium Bromide on Airway Inflammation and Programmed Cell Death 5 in a Mouse Model of Ovalbumin-Induced Allergic Asthma

**DOI:** 10.1155/2019/6462171

**Published:** 2019-10-01

**Authors:** Juan Wang, Xiaolin Diao, Hong Zhu, Bei He

**Affiliations:** Department of Respiratory and Critical Care Medicine, Peking University Third Hospital, Beijing 100191, China

## Abstract

**Rationale:**

We previously demonstrated increased expression of programmed cell death 5 (PDCD5) in asthmatic patients and ovalbumin-induced allergic asthma. International guidelines (GINA 2019) have included the use of tiotropium bromide for chronic treatment of the most severe and frequently exacerbated asthma in patients ≥6 years old, who do not have good response to inhaled corticosteroids.

**Objective:**

To explore the role of tiotropium and its effect on PDCD5 level in a mouse model of chronic asthma.

**Methods:**

We divided 12 female mice into 2 groups: untreated asthma (*n* = 6) and tiotropium-treated asthma (*n* = 6). The impact of tiotropium was assessed by histology of lung tissue and morphometry. Pulmonary function was tested by using pressure sensors. The number of cells in bronchoalveolar lavage fluid (BALF) was detected. Levels of PDCD5, active caspase-3, and muscarinic acetylcholine receptors M2 (ChRM2) and M3 (ChRM3) were examined.

**Results:**

Tiotropium treatment significantly reduced airway inflammation and remodeling in asthmatic mice and intensified the lung function. PDCD5 level was reduced with tiotropium (*p* < 0.05). Moreover, active caspase-3 level was decreased with tiotropium (*p* < 0.001), and ChRM3 level was increased.

**Conclusions:**

Tiotropium treatment may alleviate the pathological changes with asthma by regulating apoptosis.

## 1. Introduction

Allergic asthma is a major health concern worldwide, with chronic inflammatory disorder of the airways and airway remodeling. Airway remodeling can lead to irreversible airflow limitation and accelerate lung function decline [1]. Despite advances in the pathogenesis and therapeutics for asthma, for some patients, the disease remains uncontrolled without good response to inhaled corticosteroids. Novel treatment strategies need to be explored.

Tiotropium bromide (tiotropium), a selective long-acting, muscarinic acetylcholine receptor (mAChR) antagonist, is important for treating chronic obstructive pulmonary disease (COPD). Anticholinergic drugs relax airway smooth muscle by blocking mAChRs in the airway and are thus considered an alternative bronchodilator therapeutic option for asthma. Clinical evidence revealed that tiotropium can reduce severe asthma relapse [2]. In Europe and the United States, tiotropium is approved for patients ≥6 years old and with asthma uncontrolled by medium- to high-dose inhaled corticosteroids/long-acting *β*2-agonists according to the Global Initiative for Asthma (GINA) 2019 Steps 4 and 5 with a history of exacerbations [3, 4]. Evidence from mouse models show that tiotropium can suppress inflammation and airway remodeling in chronic asthma [5–8]. However, the underlying mechanism is still unclear.

Increasing evidence has shown that changes in programmed cell death or apoptosis mechanisms of resident and mobile cells of the airways may directly contribute to the development and clinical severity of asthma [9]. Apoptosis is also an important process in ameliorating inflammation.

Programmed cell death 5 (PDCD5) is a strong candidate of apoptosis-regulating proteins because of its known role in programmed cell death [10]. PDCD5 was reported to be associated with accelerated apoptosis in response to various stimuli [11]. Moreover, caspases are essential for apoptosis. Caspase-3 is considered the key executioner caspase in apoptosis [12]. We previously reported increased serum PDCD5 level in asthmatic patients. In addition, we found PDCD5 upregulated in bronchoalveolar lavage fluid (BALF) and lung tissue of ovalbumin- (OVA-) challenged asthmatic mice as compared with controls [13, 14]. Finally, the expression of PDCD5 was correlated with asthma severity and active caspase-3 levels.

In the present study, we established a mouse model of allergic asthma to investigate the effect of tiotropium on the change in asthmatic pathology and the expression of PDCD5.

## 2. Methods

### 2.1. Mice and Reagents

The study was performed in strict accordance with the recommendations in the Guide for the Care and Use of Laboratory Animals of the US National Institutes of Health. The animal protocol was approved by the Committee on the Ethics of Animal Experiments of Peking University Health Science Center, Beijing (Permit No. LA2011-062). Twelve BALB/c mice (female, 6–8 weeks old) were obtained from the Department of Laboratory Animal Science (Peking University Health Science Center). They were kept under pathogen-free conditions and had free access to food and water during experiments. Mice were randomly divided into 2 groups (*n* = 6 each): OVA-challenged group (untreated asthma group) and OVA-challenged tiotropium-treated group (tiotropium-treated asthma group).

Chicken egg OVA and aluminum hydroxide powder were from Sigma-Aldrich (St Louis, MO, USA). Tiotropium powder for inhalation (Spirva) was a gift from Boehringer Ingelheim (Ingelheim, Germany). Periodic acid-Schiff staining (PAS) and Masson's trichrome staining (Masson) kits were from Shanghai Yuanye Bio-Technology. The home-made mouse anti-PDCD5 monoclonal antibody and the PDCD5 ELISA kit were gifts from Prof. Yingyu Chen (Center for Human Disease Genomics, Peking University, Beijing). The antiactive caspase-3 antibody was from Abcam (Cambridge, MA, USA). Mouse anti-mAChR M2 (ChRM2) and anti-mAChR M3 (ChRM3) antibodies were from Shanghai Gongshuo Bio-Technology.

### 2.2. Induction of Allergic Asthma Mouse Model and Treatment Protocols

The modified OVA inhalation method was used to generate the allergic asthma mouse model as described in [14]. In brief, OVA sensitization involved an intraperitoneal injection of 20 *μ*g OVA absorbed with 2.25 mg aluminum hydroxide gel on days 1 and 14. On day 21, mice were placed in an acrylic box (40 × 30 × 15 cm) connected to an ultrasonic nebulizer (model YC-Y800, Yadu, Beijing). The untreated asthma group was challenged by repeated inhalation with 30 ml OVA (2.5% weight/volume diluted in physiological saline) for 30 min/day on 3 consecutive days/week for up to 8 weeks. For tiotropium treatment, mice were sensitized and challenged as for the untreated asthma group and then from day 21 were treated with tiotropium (36 *µ*g dissolved in 3 ml sterile physiological saline) with a nebulizer for 5 min before each OVA challenge. Mice were killed 24 h after the last exposure.

### 2.3. Pulmonary Function Measurement

Mice were anesthetized with 10% urethane injected intraperitoneally and were intubated endotracheally by using trocars. Pulmonary function was assessed by using an animal ventilator (ADInstruments, Australia) connected to a pressure sensor. The peak expiratory flow (PEF), peak inspiratory flow (PIF), intra-airway pressure (IP), and maximum rising slope of IP (IP slope) were detected, and data were analyzed by using Chart 4.1 (ADInstruments, Australia).

### 2.4. Bronchoalveolar Lavage Fluid (BALF) Cytology

The mouse lungs were successively lavaged three times with 0.5 ml physiological saline. Recovered BALF was pooled and centrifuged (2000 rpm, 5 min). BALF cells were pelleted and stained with Wright–Giemsa. A differential count of 200 cells was performed. The supernatant was stored at −80°C.

### 2.5. Histopathology Study of the Lungs

The lavaged lungs were inflated with 10% formalin and immersed in 10% formalin fixation solution. Fixed lung tissues were embedded in paraffin, and sections were stained with hematoxylin and eosin (H&E) for inflammatory cell infiltration and proliferation of smooth muscle, Masson's trichrome for airway fibrosis and collagen deposition, and periodic acid-Schiff (PAS) for goblet cells. An expert respiratory pathologist blinded to treatment groups graded the extent of inflammation in the lungs according to a semiquantitative scoring system [14].

### 2.6. ELISA

To measure the content of PDCD5 in supernatants of BALF, supernatants (100 *μ*l/well) were added to 96-well ELISA plates and incubated for 60 min at 37°C. After washing, 1 *μ*g/ml anti-PDCD5 antibody (100 *μ*l/well) was added and incubated for 60 min at 37°C. After three washes, TMB solution (100 *μ*l/well) was added for incubation in the dark at room temperature for 15 min. Color development was stopped by adding 2 M H_2_SO_4_ (50 *μ*l), and absorbance was measured at OD 450 nm (OD_450_).

### 2.7. Immunohistochemistry (IHC)

IHC was used to detect the expression of PDCD5, active caspase-3, ChRM2, and ChRM3 in the lung. Paraffin sections of 4 *μ*m lung tissue were stained with antibodies for PDCD5 (1 : 300), active caspase-3 (1 : 100), and ChRM2 and ChRM3 (1 : 100) by incubating overnight at 4°C. Secondary staining with a goat antimouse antibody involved using an ABC kit and DAB (DAKO, Carpinteria, CA, USA).

### 2.8. Statistical Analysis

Data are presented as mean ± SD. One-way ANOVA was used to compare multiple samples and the Student's independent *t* test to compare two groups. Pearson correlation coefficient was used for correlation analysis. *p* < 0.05 was considered statistically significant. Data were analyzed by using SPSS 13.0 and GraphPad Prism 5.0.

## 3. Results

### 3.1. Tiotropium Treatment Enhanced Pulmonary Function

Tiotropium treatment enhanced PIF from 2.26 ± 0.03 L/s in the untreated asthma group to 2.29 ± 0.01 L/s in the tiotropium-treated asthma group and PEF from 4.66 ± 0.04 L/s to 4.80 ± 0.12 L/s (*p* < 0.05). The IP slope decreased from 96.02 ± 3.69 mmHg/s in untreated asthmatic mice to 74.90 ± 4.90 mmHg/s in tiotropium-treated asthmatic mice (*p* < 0.001) (Table 1).

### 3.2. Tiotropium Treatment Attenuated Chronic Airway Inflammation and Airway Remodeling

After tiotropium treatment, the total number of inflammatory cells in BALF of asthmatic mice (35.92 ± 9.05 × 10^4^) showed a decreasing trend without statistical significance (27.0 ± 3.97 × 10^4^) (Table 1).

Histological staining revealed decreased inflammatory cell infiltration in airways and pulmonary vasculature, goblet cell hyperplasia, smooth muscle cell proliferation, peribronchial fibrosis, and collagen in tiotropium-treated asthmatic mice (*p* < 0.05; Table 1).

### 3.3. Tiotropium Treatment Reduced PDCD5 Level

**Figure 1:**
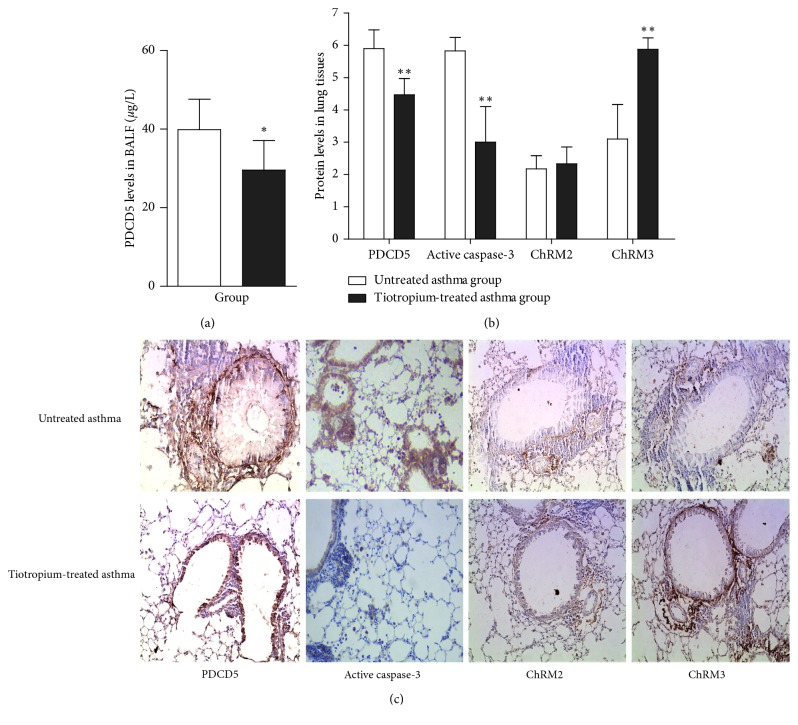
Protein expression. (a) PDCD5 protein level in BALF. (b) Protein levels in lung tissue. (c) Representative immunohistochemical staining of PDCD5, active caspase-3, and muscarinic acetylcholine receptors M2 (ChRM2) and M3 (ChRM3). Data are mean ± SD. ^*∗*^*p* < 0.05 and ^*∗∗*^*p* < 0.01 compared with untreated asthma mice.

To determine whether PDCD5 was affected by treatment, we tested PDCD5 in mouse BALF and lung tissue. The PDCD5 protein level was higher in BALF of untreated than tiotropium-treated asthmatic mice (39.89 ± 7.74 vs. 29.58 ± 7.49 *μ*g/L, *p* < 0.05) (Table 2) (Figure 1(a)). On IHC, PDCD5 protein staining was reduced in airway epithelial and inflammatory cells after tiotropium treatment (5.90 ± 0.58 vs. 4.47 ± 0.50, *p* < 0.01) (Figures 1(b) and 1(c)). In lung tissue, PDCD5 staining intensity was positively correlated with scores for inflammatory cell infiltration, goblet cell metaplasia, and collagen deposition (*p* < 0.01) (Table 3).

### 3.4. Tiotropium Treatment Reduced Active Caspase-3 Level

IHC revealed decreased active caspase-3 level after tiotropium treatment (5.83 ± 0.41 vs. 3.00 ± 1.10, *p* < 0.001) (Figures 1(b) and 1(c)). Moreover, active caspase-3 protein level was positively correlated with scores for inflammatory cell infiltration, goblet cell metaplasia, collagen deposition, and total cell number in BALF (*p* < 0.05) (Table 3). PDCD5 and active caspase-3 levels were positively correlated (*r* = 0.862, *p* < 0.001).

### 3.5. Elevated ChRM3 Level in Lung Tissues with Tiotropium Treatment

On IHC, ChRM2 and ChRM3 were expressed mainly in the airway mucosa (including airway epithelial cells and goblet cells), smooth muscle layer, and inflammatory cells around the airway. ChRM3 level was higher with tiotropium treatment than without tiotropium treatment (5.88 ± 0.35 vs. 3.10 ± 1.07, *p* < 0.001), with no difference in ChRM2 level with and without tiotropium treatment (2.33 ± 0.52 vs. 2.17 ± 0.41, *p* > 0.05) (Figures 1(b) and 1(c)).

## 4. Discussion

Our previous studies reported increased serum PDCD5 level in asthmatic patients and upregulated PDCD5 in BALF and lung tissue of untreated asthmatic mice versus controls, which was correlated with asthma severity [13, 14]. In the present study, we established a mouse model of chronic allergic asthma and diminished the severity of asthma by treatment with tiotropium. Tiotropium treatment improved the lung function of asthmatic mice with relief of airway inflammation and remodeling, accompanied by downregulated PDCD5 and active caspase-3. Tiotropium may be beneficial therapeutically as a bronchodilator and an anti-inflammatory agent by regulating apoptosis.

Although tiotropium is an anticholinergic bronchodilator commonly used to treat COPD, novel pharmacological strategies suggest its benefit as an anti-inflammatory drug [15, 16]. Clinical research proposed the use of tiotropium as an alternative treatment for asthmatic patients ≥6 years old with uncontrolled asthma because its effects appeared equivalent to salmeterol [17, 18]. However, only a few publications exist on the therapeutic effect of tiotropium in animal models of asthma, suggesting its anti-inflammatory effects on allergic airway inflammation besides bronchodilation [5–8, 19].

OVA-sensitized mice can show pathological and clinical features similar to those observed in human chronic asthma. In accordance with previous studies, we found that tiotropium might attenuate airway inflammation and airway remodeling, thus diminishing the severity of asthma. The inflammatory cell number was decreased in airways and pulmonary vasculature with tiotropium treatment, and goblet cell hyperplasia, smooth muscle cell proliferation, peribronchial fibrosis, and collagen deposition were decreased. Nevertheless, tiotropium has been found to inhibit airway resistance and compliance in asthmatic mice [6]. In agreement with this result, PIF and PEF were enhanced after tiotropium treatment in our study, which implies that tiotropium can enhance the pulmonary function of asthmatic mice. However, in disagreement with most studies and our histopathological results, we found no significant difference in BALF cytology, although total cell count and eosinophil percentage were decreased. Similarly, in a guinea pig model of chronic asthma, hematoxylin and eosin results showed that only airway eosinophilia in the submucosa of cartilaginous airways was reduced by tiotropium, with no difference in airway eosinophilia in adventitia of cartilaginous airways after tiotropium treatment or in the submucosa and adventitia of noncartilaginous airways [20]. The possible reason is that being less invasive, BALF may not be enough to reflect differences in this study.

Much evidence has shown that dysregulated cell apoptosis may play a central role in the development of airway inflammation in asthma [21]. The process of apoptosis is important because it allows for rapid clearance of senescent or damaged cells, for limited tissue injury. PDCD5 was upregulated in cells undergoing apoptosis. In this study, PDCD5 expression in asthmatic airway epithelium and inflammatory cells was decreased when tiotropium relieved airway inflammation and remodeling. In agreement with our previous results, the level of PDCD5 was correlated with the lung function, inflammatory cell infiltration, goblet cell metaplasia, and collagen deposition. The consistent correlation between PDCD5 expression and severity of asthma indicated not only the potential role of PDCD5 in monitoring asthmatic severity but also a correlation between tiotropium and apoptosis. Tiotropium was previously suggested to affect apoptosis. In COPD patients, tiotropium was found to reduce CD4+ and increase CD8+ peripheral blood T-cell apoptosis via caspase-3 and caspase-8 activity and I*κ*B-mediated mechanisms [22]. In a subacute cigarette exposure mouse model, tiotropium significantly decreased the number of macrophages and caspase-3-labeled cells of the lungs [23]. In this study, we suggest that tiotropium might relieve airway inflammation by regulating apoptosis of airway epithelium and inflammatory cells. PDCD5 could evoke apoptosis of cells [24, 25]. The initiation of apoptosis serves to terminate the inflammatory process by reducing the number of inflammatory cells, but the persistence of inflammation may be due to abnormalities in the regulation of cell apoptosis, leading to a chronic or everlasting inflammatory cell survival and accumulation. Thus, with resolution of airway inflammation and clearance of inflammatory cells with tiotropium, PDCD5 expression was reduced. However, further study is needed to explore the effect of tiotropium on apoptosis.

Active caspase-3 is the key executioner of caspase and an early marker of apoptosis [12, 26]; therefore, we measured the level of active caspase-3 to confirm the correlation between tiotropium and apoptosis. Similar to PDCD5, activated caspase-3 was reduced with tiotropium treatment, and such downregulation was positively correlated with PDCD5 level (*p* < 0.001). Downregulated active caspase-3 indicates decreased cell apoptosis during the resolution of airway inflammation. We further identified the correlation between tiotropium and apoptosis.

Tiotropium is a selective ChRM3 antagonist that dissociates more slowly from M3 than M2 or M1 muscarinic receptors [27]. ChRM1 receptors are mainly distributed in the peripheral lung tissue and in the alveolar walls [28] and regulate cholinergic transmission [29]. ChRM2 receptors are on smooth muscle (SM) cells and fibroblasts [28]. Together with ChRM2, ChRM3 receptors are the most represented in human airways; they are predominantly expressed in SM cells and mediate SM ACh-induced contraction [28, 29]. ChRM3 is a G-protein-mediated receptor and may play an important role in the pharmacological effects of tiotropium [30, 31]. Some studies demonstrated that ChRM2 and ChRM3 receptors mediate proliferation of lung fibroblasts or SM cells, and tiotropium can inhibit such proliferation [32–34]. Considering the possible role of ChRM2 and ChRM3 receptors in tiotropium regulating apoptosis, we examined their levels in lung tissues. In lipopolysaccharide-induced lung inflammation, blockage of mAChR exerts anti-inflammatory properties, with ChRM3 receptors playing an important role by mediating NF-*κ*B signaling [35]. In murine models of COPD and asthma, ChRM3 expression was inhibited by the administration of tiotropium [36, 37]. Holownia et al. [38] found that tiotropium could increase cytosolic ChRM3 protein level in induced sputum cells of COPD patients. Similarly, ipratropium bromide, another common acetylcholine receptor antagonist, could upregulate ChRM3 expression in bronchial walls of an asthmatic murine model [39]. The results of existing studies are inconsistent. In our study, ChRM3 level was elevated after tiotropium treatment. Upregulated ChRM3 might be a compensatory result after continuous application of antagonists. No difference in ChRM2 level between the two groups may suggest that tiotropium did not affect ChRM2 level, perhaps because tiotropium dissociated faster from ChRM2 than ChRM3. These results suggest that anti-inflammatory effects rather than a cholinergic action in airway muscle may be the main role of tiotropium.

There were several limitations to this study. First, our asthmatic mice model is a chronic asthma model and not treated with steroids; thus, pathogenesis and pathological changes may not be similar to clinical refractory asthma. Recent published data suggested no significant difference in the response to mometasone or tiotropium as compared with placebo in mild persistent asthma with low eosinophil level [40]. Thus, exploring the mechanisms of tiotropium in asthma is relevant for all disease severity levels (so patient therapy should be individual and probably more effective in low eosinophilic asthma). Second, we did not perform in-depth research of the pathogenesis of severe asthma. Third, the molecular mechanism of the effect of tiotropium on apoptosis was not studied. Treating airway inflammatory cells with tiotropium may help explore the underlying mechanism.

## 5. Conclusions

Taken together, we found that tiotropium could improve the lung function and reduce PDCD5 level in OVA-induced asthmatic mice. Tiotropium may relieve clinical pathological changes by regulating apoptosis in asthma. Future studies are needed to determine the role of tiotropium in asthma.

## Figures and Tables

**Table 1:** Characteristics of untreated asthma mice and tiotropium-treated asthma mice.

Characteristics	Untreated asthma mice	Tiotropium-treated asthma mice	*p* value
*Pulmonary function*			
Peak inspiratory flow (L/s)	2.26 ± 0.03	2.29 ± 0.01	0.030
Peak expiratory flow (L/s)	4.66 ± 0.04	4.80 ± 0.12	0.034
Intra-airway pressure (IP, mmHg)	2.38 ± 0.06	2.33 ± 0.06	0.182
Maximum rising slope of IP (mmHg/s)	96.02 ± 3.69	74.90 ± 4.90	<0.001

*BALF cytology*			
Total cell count (×10^4^)	35.92 ± 9.05	27.00 ± 3.97	0.064
Macrophages (%)	67.00 ± 4.86	66.50 ± 3.73	0.846
Eosinophils (%)	11.50 ± 3.02	10.50 ± 1.87	0.509
Neutrophils (%)	10.67 ± 2.50	11.5 ± 1.87	0.530
Lymphocytes (%)	10.83 ± 1.47	11.50 ± 2.17	0.549

*Pathological scores*			
Inflammatory cell infiltration	2.50 ± 0.84	1.25 ± 0.42	0.013
Goblet cell hyperplasia	1.92 ± 0.58	1.17 ± 0.41	0.030
Collagen deposition	1.42 ± 0.38	0.67 ± 0.26	0.003

Data are mean ± SD.

**Table 2:** Programmed cell death 5 (PDCD5) level in BALF and lung tissues in mouse groups.

PDCD5	Untreated asthma mice	Tiotropium-treated asthma mice
BALF (*μ*g/L)	39.89 ± 7.74	29.58 ± 7.49^*∗*^
Lung tissue	5.90 ± 0.58	4.47 ± 0.50^*∗∗*^

Data are mean ± SD. ^*∗*^*p* < 0.05 and ^*∗∗*^*p* < 0.01 compared with untreated asthma mice.

**Table 3:** Correlation between PDCD5/active caspase-3 level and various clinicopathologic indexes (*n* = 12).

Protein level in lung tissue		Inflammatory cell infiltration	Goblet cell metaplasia	Collagen deposition	Total cell number in BALF
PDCD5	*r*	0.904	0.814	0.773	0.258
	*p* value	<0.001	0.001	0.003	0.418

Active caspase-3	*r*	0.754	0.681	0.739	0.625
	*p* value	0.005	0.015	0.006	0.030

BALF: bronchoalveolar lavage fluid.

## Data Availability

The data used to support the findings of this study are included within the article.
